# The method of reflection-based marker detection and identification to ensure accurate AGV docking

**DOI:** 10.1038/s41598-025-25357-x

**Published:** 2025-11-21

**Authors:** Piotr Biernacki, Adam Ziebinski

**Affiliations:** 1 Institute of Theoretical and Applied Informatics, Polish Academy of Sciences, 44-100 Gliwice, Poland; 2 Department of Distributed Systems and Informatic Devices, Silesian University of Technology, 44-100 Gliwice, Poland

**Keywords:** 2D LiDAR, 2D marker, object detection, object identification, AGV docking, Engineering, Computer science

## Abstract

Accurate localization is essential for Automated Guided Vehicles (AGVs) to ensure reliable motion planning and precise execution of docking tasks. A key challenge lies in robust environmental perception for industrial applications. This paper introduces a novel reflection-based marker detection and identification method that relies solely on two-dimensional Light Detection and Ranging (2D LiDAR) technology. The proposed docking method and 2D marker design enable the AGV to accurately estimate the marker’s distance and orientation, reliably identify it, and determine the docking point. Experimental validation on a heavy industrial AGV demonstrated that the docking method achieves accuracy of up to 1 cm in position and below 0.05 degree in YAW orientation. As a result, the AGV achieved docking precision at an assembly station with a standard deviation below 2 cm in X and Y axes and YAW orientation below 1.8 degree.

## Introduction

Current intelligent production systems require autonomous decision-making to monitor and support the production processes, especially in internal logistics systems. Internal transport systems (ITS)^1^ are increasingly using Automated Guided Vehicles (AGVs)^2^ to cooperate with warehouse systems, automatic production loading stations, robot stations^3^ or collaborative robots^4^ as well as with charging stations^5^. The movements of AGVs must be monitored^6^ and performed in a dynamic, autonomous and safe manner^7^. Therefore, AGVs are often built using distributed real-time control architecture^8^ that is equipped with different actuators and sensors, e.g., ultrasound, infrared^9^, LiDAR Sensor (Light Detection and Ranging), IMU^10^, a camera, Ultra-Wideband (UWB)^11^, encoders or even additional passive wheels^12^.

The various integrated distance sensors enable the increased determination of an AGV’s position using different localisation methods^13^. This enables them to monitor and perform precise movements in accordance with the orders that are issued by the logistics system. The positioning methods that are used by the navigation systems enable the implementation of autonomous driving methods^14^. Additionally, in order to perform logistic tasks and ensure safety in a changing industrial environment^15^, it is necessary to equip AGVs with an object detection functionality^16^. Using this technology enables AGVs to perform their tasks autonomously by identifying objects and determining their position.

Precisely determining the position^17^ of an AGV enables the navigation system^18^ to give orders correctly and increases the accuracy of the movement of an AGV. The quality of the data that is measured from the sensors has a major impact on the accuracy of the position determination of an AGV^19^. Sensors usually perform measurements in accordance with the described technical parameters. The sensor measurement error is usually sufficient when an AGV is moving in a free space. However, it may be important when the AGV has little space to avoid obstacles or needs to pass through narrow places. Additionally, it has an influence on the quality of object identification. In this case, it is important to determine the exact location correctly and to recognise any objects in the vicinity of an AGV. Using different calibration methods^20^ for the cameras^21^ and LiDARs^22^ enables the measurement error to be reduced.

In order to detect objects including their localisation^23^ and identification^24^, it is necessary to define a model of an object that contains a set of features that characterise it such as size, shape, structure and colour. Current location and tag identification systems mainly use camera-based object identification methods^25^. Excellent results in this aspect are obtained using stereo cameras with depth measurement, which enable objects to be recognised in three dimensions^26^. The quality of the measurements, however, drops considerably when the illumination intensity of the object to be recognised is low.

In order to apply the position determination functionality, it is necessary to be able to measure the distance and angle in relation to the detected object^27^. Modern AGVs mainly use LiDAR technology for this. Two-dimensional Light Detection and Ranging (2D LiDAR) systems can measure the distance, angle and reflectance intensity for a given measurement point that is illuminated by pulses of light from a laser. As a result, these properties can also be used to identify and determine the location of objects. The disadvantage of 2D LiDAR solutions is that objects and their features can only be detected and analysed in the two dimensions of the Cartesian coordinate system, which enables to determine, e.g., the length and depth of an object. On the other hand, the advantages of these systems are that measurements can be taken in low-light conditions and that a higher measurement accuracy can be achieved compared to visual solutions. As a result, a LiDAR system can be used to locate and identify objects in an industrial environment. In effect, it can be used to precisely dock an AGV to an assembly station (AS).

The aim of this paper is to illustrate the potential of using 2D LiDAR to identify and locate objects in industrial environments. The main challenge was to develop a method for reflection-based marker detection and identification using 2D LiDAR technology. The developed 2D marker and the method for its detection and identification enables to determine the relative position and orientation of an AGV to the detected marker. Additionally, a 2D marker enables to embed an information due to the differences in the reflectivity of a marker’s surface, which enables to identify a marker. Even a single marker can be used to determine its position and orientation relative to an AGV.

The efficiency of the developed method was validated by docking experiments that were conducted using an industrial AGV system. After docking the developed 2D marker and the method were used to accurately verify the position of a docked AGV.

The novelty of the proposed solution is the use of only 2D LiDAR to identify and locate objects in an industrial environment. The presented method will facilitate the more accurate verification of the designated route, the precise docking of AGVs to the AS, and thus, the correct realisation of logistical tasks. It is essential to update the docking point against properly set markers and to verify the position of an AGV with a high degree of accuracy during the docking process. This is a fundamental step in ensuring the correct and repeatable operation of the other automated, robotic and collaborative robot subsystems^28^.

## Related works

In response to the demand for vision tags in logistics systems, many types of markers^29^ are now available for detecting, tracking and identifying objects which are in different shapes: square (ARTags, AprilTag, ArUco, QRcode), round (ShotCode)^30^ and rectangular (linear and 2D barcodes). Tags should be robustly identifiable and distinguishable to the vision system^31^.

Many autonomous solutions require the very precise movement of a robot or vehicle^32^, and therefore, markers need to be detectable while in motion, at the required distances and under varying lighting conditions. In addition, they should provide an accurate estimate of the 6D position^25^ (3D position and 3D orientation) of the marked object in the observed scene. A marker is located by locating an object in buildings or in an open space to which the marker is attached in an appropriate manner according to the standard. For example, tagged objects are various objects, vehicles, robots, flying systems, infrastructure etc. The main components of the identification and localisation system are electronic control units (ECUs) that are informationally coupled to a vision solution that are equipped with a mono- or multi-camera system or a 2D or 3D LiDAR system that are carried on robotic mobile platforms (e.g., various types of vehicles, guided systems or autonomous systems, e.g., AGVs, UGVs and UAVs).

**Figure 1:**
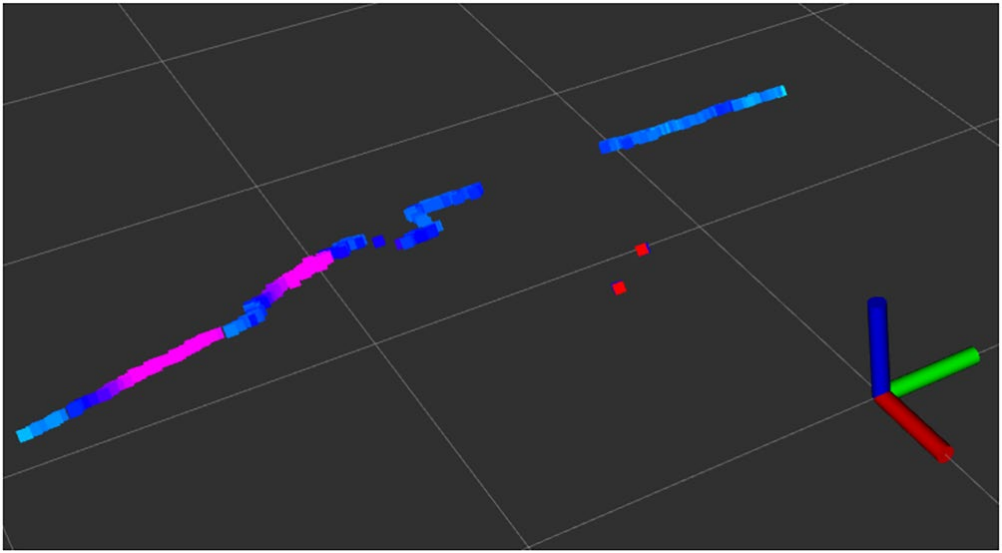
Example of a 2D LiDAR scan with the information about the strength of the reflected laser beams.

Autonomous logistics solutions in industrial environments mainly use LiDAR technology for AGVs for this purpose. It enables the accurate measurement of distance and angle in relation to the observed object, which is essential to accurately estimate their position in navigation systems. In addition, based on the reflection intensity (strength of a light wave reflectance) for a given measurement point that is illuminated by pulses of light from a laser, some LiDAR types enable the measurement of the level of the reflected laser light (Fig. 1). This property can be used in a variety of applications. For example, it is possible to automatically extract road markings from mobile LiDAR point clouds^33^.

To date, 1D, 2D and 3D tag identification systems that use various types of LEDs including infrared, scanners, lasers and cameras are available. There are many solutions that are available on the market in this area (various types of readers, e.g., barcode, 2D and 3D) and they fulfil the tag identification functionality, however, these solutions do not enable the tag location and shape identification of individual tag positions.

In the paper^34^, the authors propose a non-linear Markov switching algorithm for detecting and tracking kerbs based on information from cameras and 2D LiDAR. Paper^35^ presented a baseline clustering method for detecting cylindrical objects and a tracking system in a global coordinate frame that was based on 2D LiDAR data and RTK-GNSS positioning. The marker identification systems that are currently used mainly use object identification methods based on camera images with location support using 3D LiDAR. In^36^, the authors proposed a graph-based formula that represents a continuously minimised cost function for estimating the position and map in the SLAM of the copter. In the solution presented there, AprilTags were identified by the camera, while the position was determined by the 3D LiDAR. In^37^, the authors presented an approach for detecting an object by adapting the CNN structure to process 3D LiDAR data in a real-time driving environment. In this case, the LiDAR information was projected into their bird’s-eye view representation. From the US patent description^38^ is known a surface inspection device comprising an illumination system for focusing a beam of radiation at a non-perpendicular angle of incidence to form a line of illumination on a surface in the plane of incidence of the focused beam. It allows collection of illumination data and imaging of the laser illuminated line on an array of detectors positioned parallel to the illumination line for inspection of different types of surfaces. The above solution enables the surface to be analysed using a device that is aligned parallel to it. From another US description^39^, it is shown how to train an artificial intelligence module for an advanced driver assistance system for obtaining an in-depth model of a scene using the fusion of LiDAR, a camera and artificial intelligence module data for depth detection. An overview of 3D LiDAR technology for object detection, mainly using artificial intelligence methods, in automotive applications is presented in the articles^40^ and^41^.

As a result, the LiDAR properties enable the object’s parameters, including their length, height (3D LiDAR), depth, orientation angle and reflection level to be measured. Consequently, this makes it possible to identify and determine the position of a marker and, in effect, to identify a specific object in industrial environments. Both camera and LiDAR technologies are often used to identify object. 2D LiDAR solutions are often used in industrial logistics systems for navigation but can also be used to detect and identify objects. The problem that had to be solved was to develop a new method for processing marker identification and localisation based on a 2D LiDAR measurement system.

## The method of reflection-based marker detection of AS using 2D LiDAR

This section describes the detection and identification method of the developed marker based on the width, depth and reflection level of the LiDAR beams that are being used to identify a marker mounted on the AS. The subject of the invention is a method and a system for identifying and locating markers using a 2D LiDAR, which are particularly applicable for the identification and location of objects that have been tagged with markers. 2D LiDARs provides information about the distance to an obstacle from which the beam was reflected at specific angle as well as information about the strength of the reflected beam.

**Figure 2:**
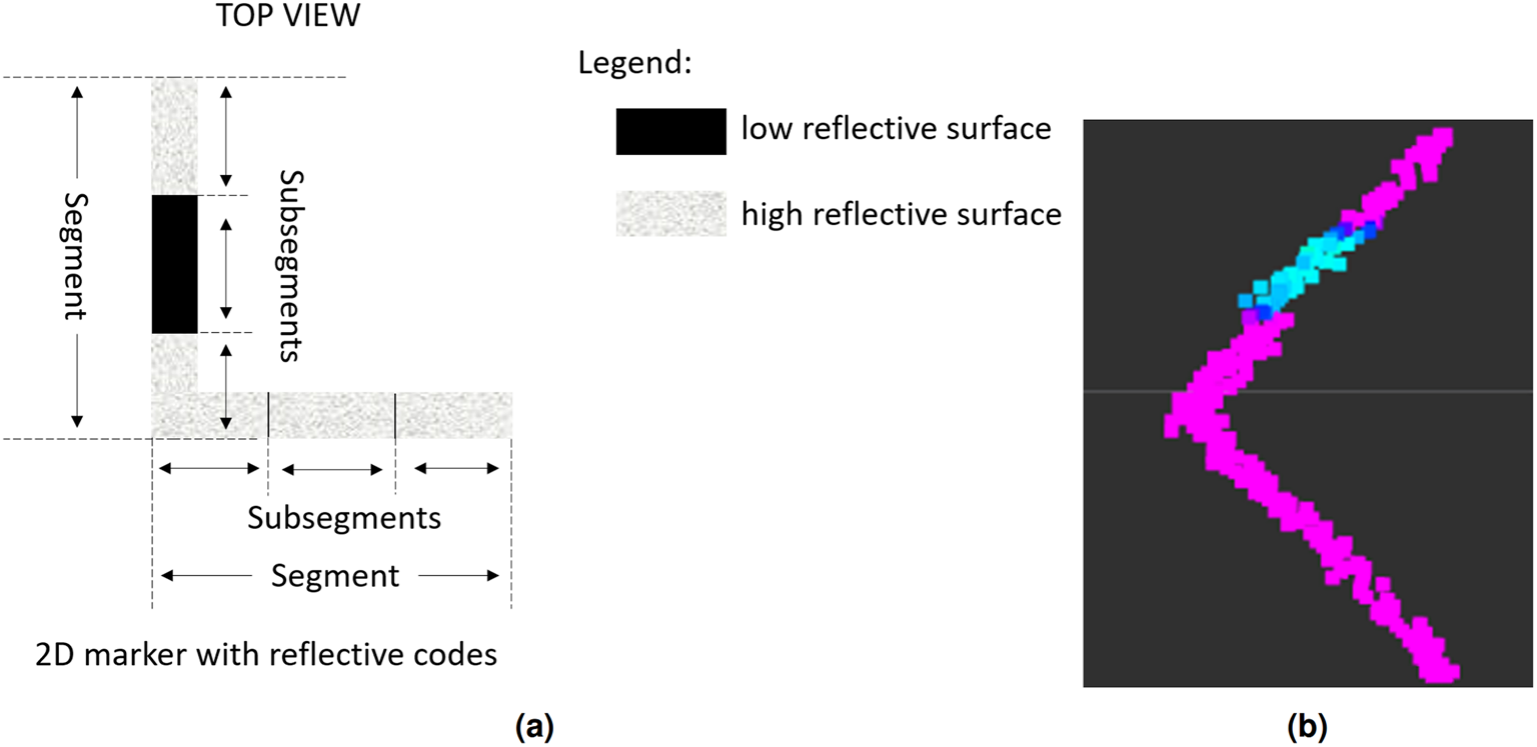
Example of a 2D marker with reflective codes (**a**) and the laser scan that was obtained by the 2D LiDAR (**b**). Each of segments consists of reflective and non-reflective subsegments.

**Figure 3:**
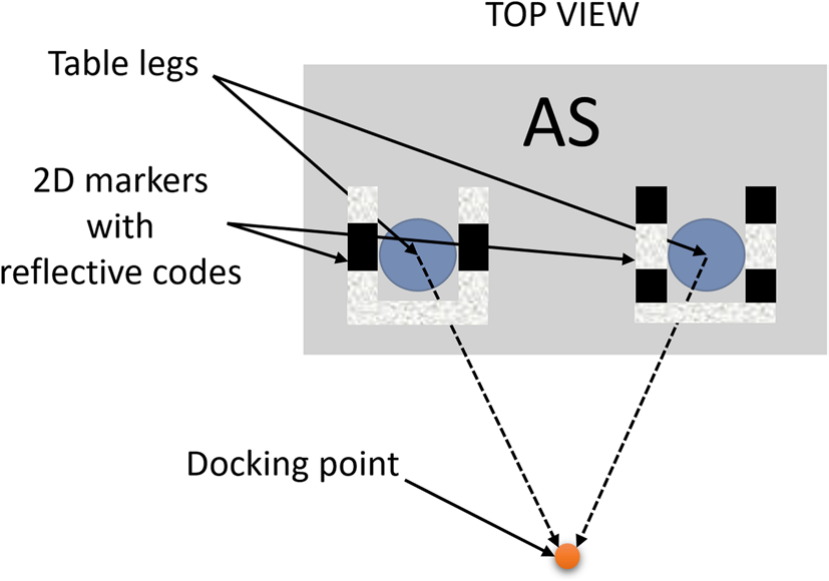
Example of 2D markers with reflective codes mounted on the legs of an AS.

The 2D markers with reflective codes (2DM-RC)s that were designed by the authors can be used for various purposes, but they were developed for accurately docking an AGV into an AS. As an example, the 2DM-RC (Fig. 2a) can consist of two straight segments placed at a specific angle, in our case, they were perpendicular to each other. Each of the segments can have a different length. The length of a segment will depend on the maximum range from which the 2DM-RC can be seen and the angular resolution of the 2D LiDAR that is used. These two parameters will define how many beams of the 2D LiDAR will be able to reflect from the segments of the 2DM-RC.

The perpendicularity of the 2DM-RC that were designed for docking enable the relative orientation of the AGV against the AS to be determined. Because of the LiDAR’s information about the strength of the reflected beam, there is a way to sense the reflectiveness of the surfaces and this enables the information in each of the segments to be embedded. Both segments of the 2DM-RC are covered partially or fully by the reflective tape, thus embedding the information on the surface of the segments. The straight segments of the 2DM-RC can be divided into subsegments, where each subsegment is covered or not by the reflective tape, thus creating the code through binarisation, which can be treated as an n-bit identifier. In our case, the 2DM-RC were mounted on the legs of the AS. The fully reflective segment of the 2DM-RC was treated as the front of the AS and the second segment of the 2DM-RC had information about which leg of the AS it was left or right (Fig. 3).

Information from 2D LiDAR about the distance and reflection strength of every beam are also embedded in the scan. The picture on the right (Fig. 2b) presents the LiDAR scan of the 2DM-RC. The pink colour indicates a highly reflective surface and the blue one as a dark and low reflective surface. Specific steps were developed to analyse the 2D LiDAR scan and to detect and identify the 2DM-RC.

**Figure 4:**
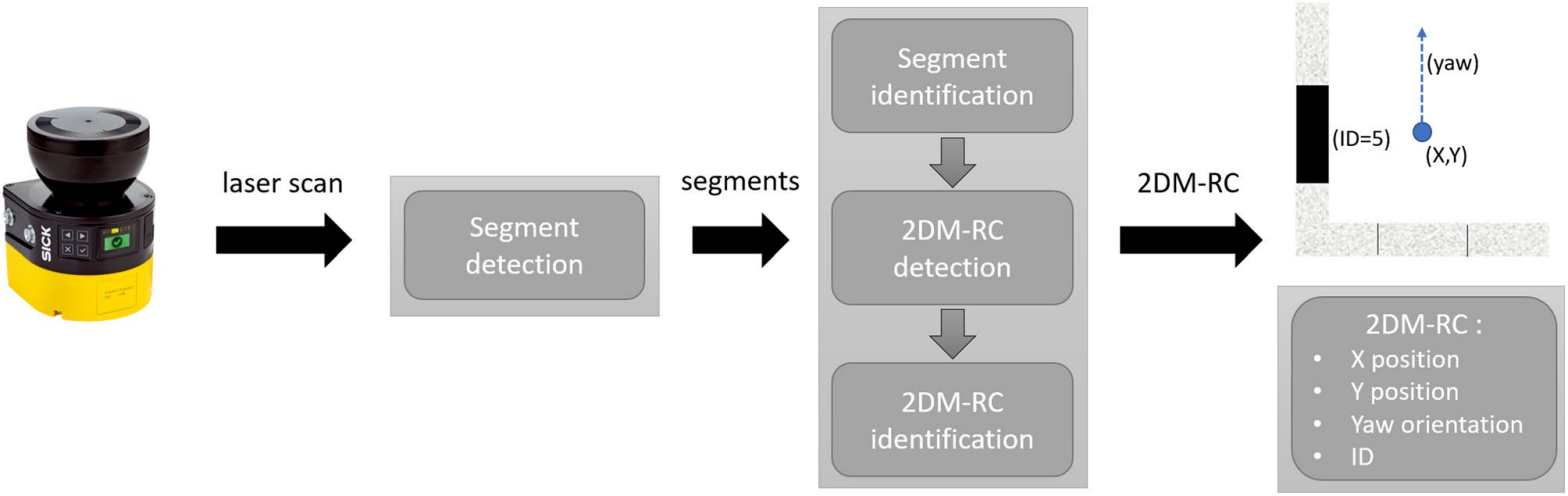
Diagram of the 2DM-RC detection and identification processing pipeline.

The method of reflection-based marker detection and identification using 2D LiDAR sensor consists of several stages segment detection, segment identification, 2DM-RC detection and 2DM-RC identification (Fig. 4). Everything begins with gathering the distance measurements and reflection information from the 2D LiDAR that will be further analysed.

The first stage is segment detection using an Iterative End Point Fit (IEPF) algorithm^42,43^. This segment detection method was developed and described in detail in^44^. At the beginning, the laser scans are converted from a polar to cartesian coordinate system, then grouped and segmented using the IEPF algorithm. The output of this stage is a set of segments, straight lines that consist of group of points with their start and end points. The authors have developed further existing segment detection solution, which will enhance the output segments with coordinates of all of the points that a segment consists of, reflection information corresponding to each point in a group, the centre position of the segment and the information about the orientation of a given segment in the coordinate system being analysed. As a result of this modification, the authors had enough information about the straight segments in the environment to develop the next stages of the 2DM-RC detection and identification method.

The second stage is the segment identification, which can be done based on their length, reflectiveness or both. The authors have focused on an identification that is based on the reflectiveness. Segments longer or shorter than the desired length are eliminated. Then, the segments that remain are identified based on the information about their reflectiveness, which is then used to binarise each of the detected segments. The identification stage depends on the information about the number of bits that are embedded in the segment. In this article, the authors focus on a 3-bit system. The segment that is being identified and the points with information about its reflectiveness are divided into three subsegments. For each of the subsegments, an mean of the three most frequent reflectiveness values is calculated. If the mean is higher than the specified threshold, the binary representation for the subsegment is 1, otherwise, it is 0. The gathered bits are then bit-shifted to form a 3-bit identifier for the entire segment (e.g., ID=7 111, ID=5 101) (Fig. 3 front and left segment). The segments with an ID that are not available in the system are eliminated.

The segments that remain move into the 2DM-RC detection stage where the mutual distance and orientation of the segments are determined. If the two segments are in the desired orientation and distance, they are classified as 2DM-RC. Then, the exact position of the AS leg is calculated based on the detected 2DM-RC that is mounted on the leg of the AS. Knowing the start and end points of the segments, the vector is calculated. The vector is then rotated clockwise by 90 degrees, normalised and multiplied by the distance from the segment to the centre of the AS leg on which the 2DM-RC was mounted. This vector is then added to the middle point of the segment, which determines the centre of AS leg.

The final stage is the 2DM-RC identification in which the combination of the information that is embedded in the segments determines the ID of the entire 2DM-RC. The detected 2DM-RC is analysed for the IDs of the segments which it consists of. It was assumed that the AS leg had to consist of two segments where one of them is fully reflective (ID=7 111, denotes front side of AS) and the second one contains information about the side (right or left leg, ID=5 or ID=2). This information is crucial for correctly calculating the docking point.

To summarise, the 2D LiDAR scan analysis and the 2DM-RC detection and identification method consists of the following steps:

1. Laser scan acquisition
  - distance and reflected laser beam strength measurements are gathered from the 2D LiDAR.
2. Laser scan to Point Cloud (PCL) conversion
  - the X, Y coordinates are assigned for each laser beam point,
  - conversion from a polar to a cartesian coordinates system.
3. Segment detection
  - segments consisting of the PCL points are detected.
4. Segment filtration by its length
  - every detected segment longer or shorter than the specified length is eliminated and no longer processed.
5. Segment identification
  - every segment is divided into subsections which are binarized (0/1) depending on the value of the intensity of the dominant beams and the threshold value,
  - the decoded ID is assigned to a specific segment.
6. Segment filtration by its ID
  - every segment with an ID other than those permitted is eliminated and no longer processed.
7. The 2DM-RC detection
  - all of the remaining segments are checked for mutual distance and orientation,
  - segments with desired mutual distance and orientation become 2DM-RCs,
  - the position and orientation are calculated for each detected 2DM-RC.
8. The 2DM-RC identification
  - The combination of the information that is embedded in the segments determines the ID of the whole 2DM-RC, thus its purpose or meaning in docking process.

## Development of methods for docking an AGV to an AS

Precise docking of an AGV to an AS enables the correct performance and completion of the production process. Therefore, the docking procedure for an AGV to the assembly line plays an important role, especially in the solutions for the processes that are used in Industry 4.0. The methodology for docking algorithms and the verification of an AGV’s position and orientation relative to an AS after docking employs a reflection-based marker detection and identification approach utilizing 2D LiDAR. This approach enables the quality of the docking algorithm to be verified and ensures the exact position of the AGV in relation to the AS even if errors were made during the docking.

The proposed methods enable the precise docking of an AGV to an AS. An application of this method was presented on the example of the docking experiments of the industrial AGV called a CoBotAGV “Formica 3”^28^. In order to perform precise docking, a CoBotAGV needs to first locate and identify specific elements of an AS in the surrounding environment and drive to a certain point in front of an AS. The 2DM-RCs are identified using 2D laser safety scanners (Sick microscan3), which are mounted on a CoBotAGV.

In order to perform precise docking, several assumptions for the docking procedure were formulated:

1. A collaborative robot will be mounted on the top of the CoBotAGV, and therefore an accuracy error should be less than 2 cm because of the limited movement area of the arm of the collaborative robot,
2. The adjustable starting point of docking should be no further than 2 m away from the nearest 2DM-RC that is mounted on an AS. The reason for that specific distance is the limited angular resolution of 2D LiDAR. In order to identify and locate the AS, there must be enough measurement points on the surface of the AS,
3. During the docking movements, the speed is limited in order to provide real-time operability in order to observe the nearest AS environment,
4. External markers can be used on the AS,
5. The available methods should include an analysis of the 2D LiDAR data,
6. The docking method should include a verification phase after the docking point is reached,
7. To perform the verification phase, the AGV must be able to see at least one 2DM-RC after the docking point is reached.

Before the docking procedure begins, the natural navigation (third-party navigation system) guides the CoBotAGV to the initial position of the docking phase. Next, the docking algorithm that is based on Robot Operating System (ROS)^45^ that was developed by the authors is activated.

**Figure 5:**
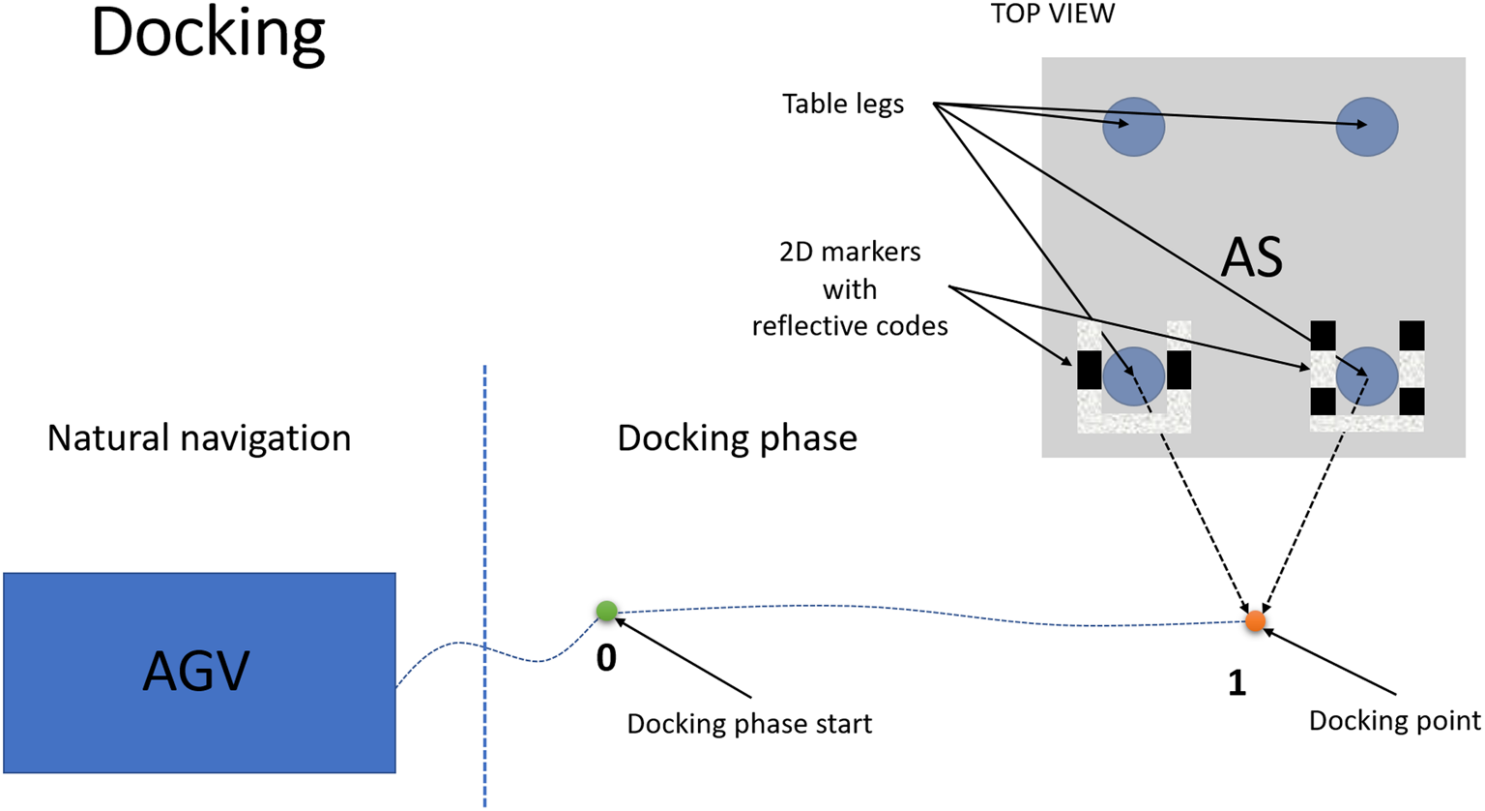
Procedure for docking an AGV to an AS equipped with 2DM-RC mounted on the legs docking phase.

**Figure 6:**
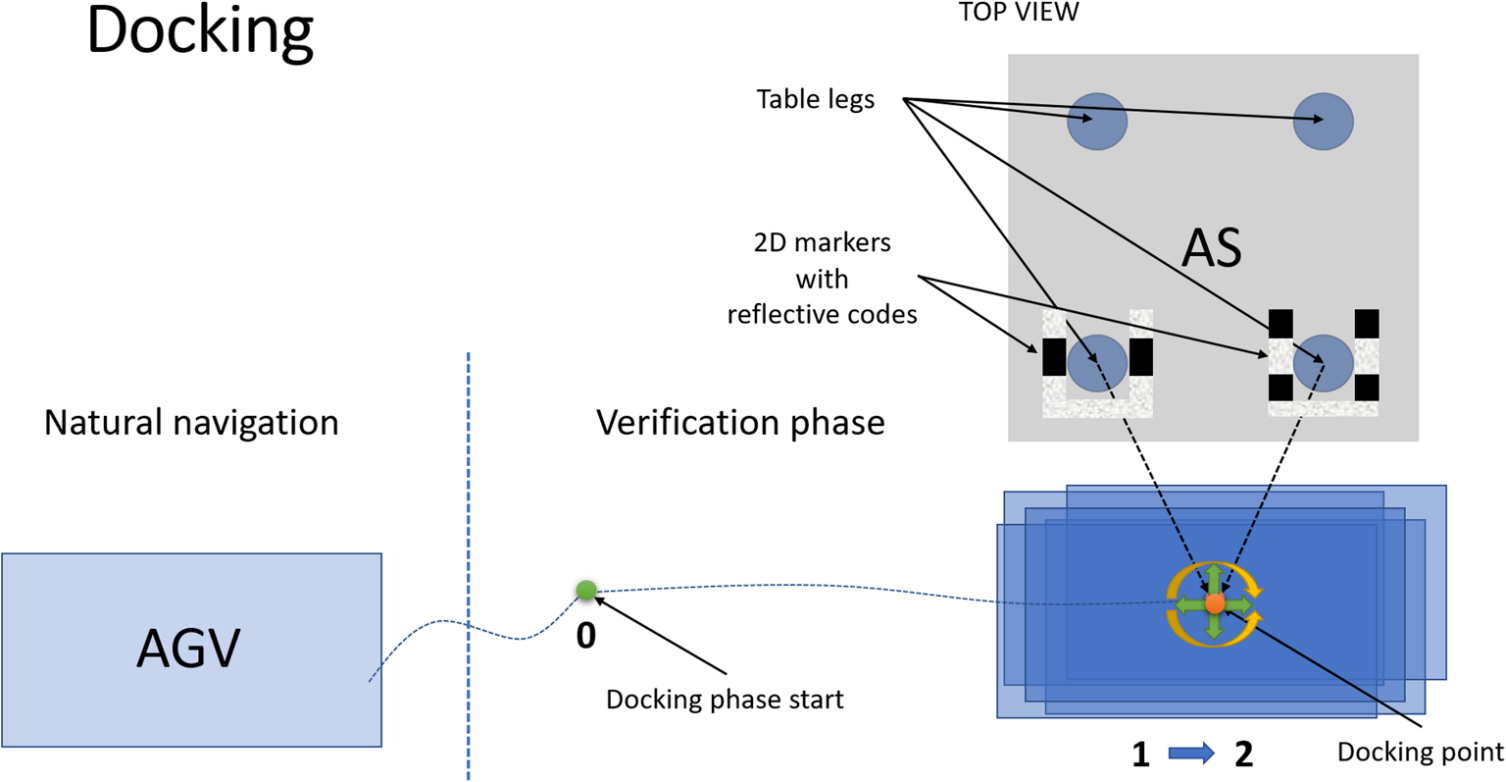
Procedure for docking an AGV to an AS equipped with 2DM-RC mounted on the legs verification phase.

The docking procedure consists of three steps:

1. Initial position (this step is illustrated in Fig. 5):
  - the CoBotAGV begins to look for known marker patterns (2DM-RCs), which enable it to locate and identify the specific elements of the AS,
  - as soon as at least one of the 2DM-RCs is identified, the docking point is calculated, and the docking phase begins.
2. Docking phase (this step is illustrated in Figs. 5 and 6). The docking point is the destination of the docking phase. It is located at a specific distance from the AS:
  - the CoBotAGV is controlled by the ROS navigation stack to the docking point,
  - as soon as docking point is reached by the ROS navigation, the docking phase ends, and the verification phase begins.
3. Verification phase (this step is illustrated in Fig. 6). The verification phase is the step during which the CoBotAGV performs an additional test to obtain the final information about the accuracy of the performed docking procedure:
  - the CoBotAGV begins to look for the known marker patterns (2DM-RCs), which enable it to locate and identify the specific elements of the AS (AS),
  - as soon as at least one of the 2DM-RCs is identified, the correction value after the docking phase is calculated. The X,Y positioning and the orientation can be inaccurate in relation to the AS.

### The method for detecting and identifying 2D reflection-based markers using 2D LiDAR

The docking procedure for a CoBotAGV is possible due to the development of 2D reflection-based markers and the methods that are used to locate and identify these markers, which are mounted on an AS elements.

In our case, the CoBotAGV needed to perform the docking procedure from the side of the AS. This meant that there would be no physical element directly in front of the CoBotAGV to which the distance could be measured and analysed further. Therefore, a special marker that could be seen by the 2D LiDAR (where single LiDAR has field of view equal to $270^\circ$) had to be created that could analyse the entire environment around the CoBotAGV.

Based on the docking assumptions, the 2DM-RC to be created had to be at least 21cm long for each of the straight perpendicular segments. This would enable enough laser beams to collide (around 40 beams having 0.1 degree resolution 2D LiDAR) with the segments of the 2DM-RC even if the 2DM-RC was turned and was seen under a 45 degree angle.

Additionally in order to differentiate segments and enable the identification of the 2DM-RCs, one of the segments had to consist of the three (equal in length) subsegments with different reflectance. This would enable the identification number (ID) to be encoded in a 3 bit binary system in which the more reflective segment is coded as 1 and the less reflective segment is coded as 0. The information about the encoded ID is connected with the detected AS leg. The strength of the reflected laser beam is used to identify and assign specific IDs to specific segments.

After discovering the required shape (2DM-RC), there is access to the X, Y points and the orientation information about the elements of the AS that was discovered. Based on this information, the position of the CoBotAGV, its orientation in relation to the AS and the distance of the CoBotAGV from the AS can be calculated.

**Figure 7:**
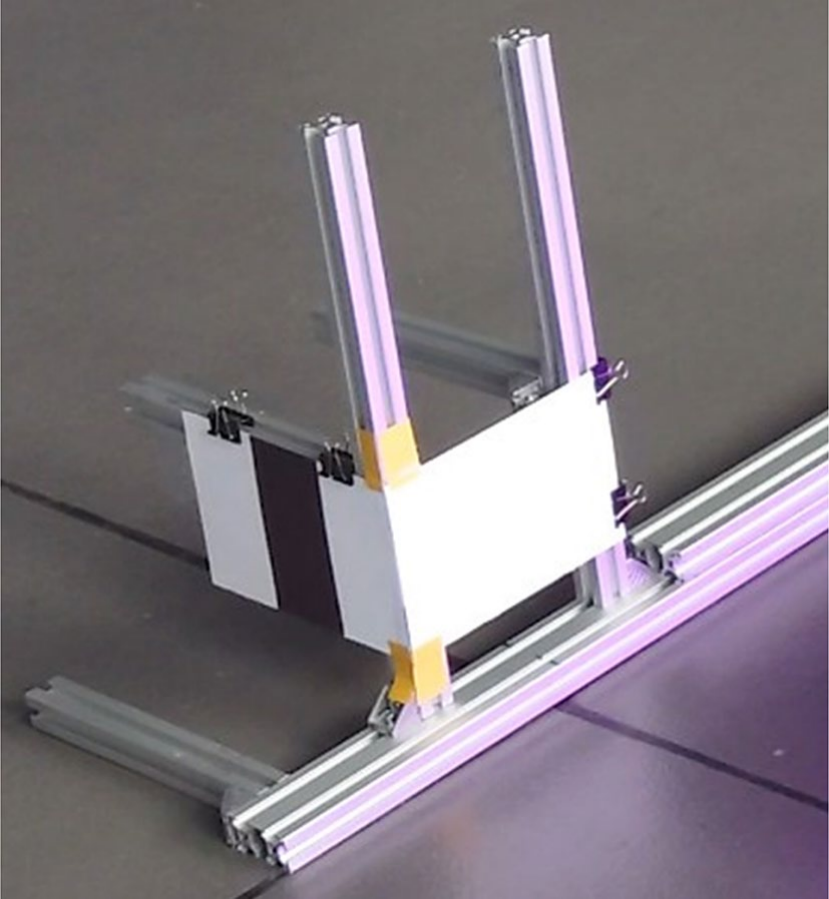
The test stand for one AS leg (left) equipped with a 2DM-RC.

Figure 7 show the custom research stand of the 2DM-RC for testing purposes, which consists of two hard white paper sheets with glued reflective tape and a dark piece of paper glued in the middle on one of them. The entire 2DM-RC was mounted on an aluminium construction.

**Figure 8:**
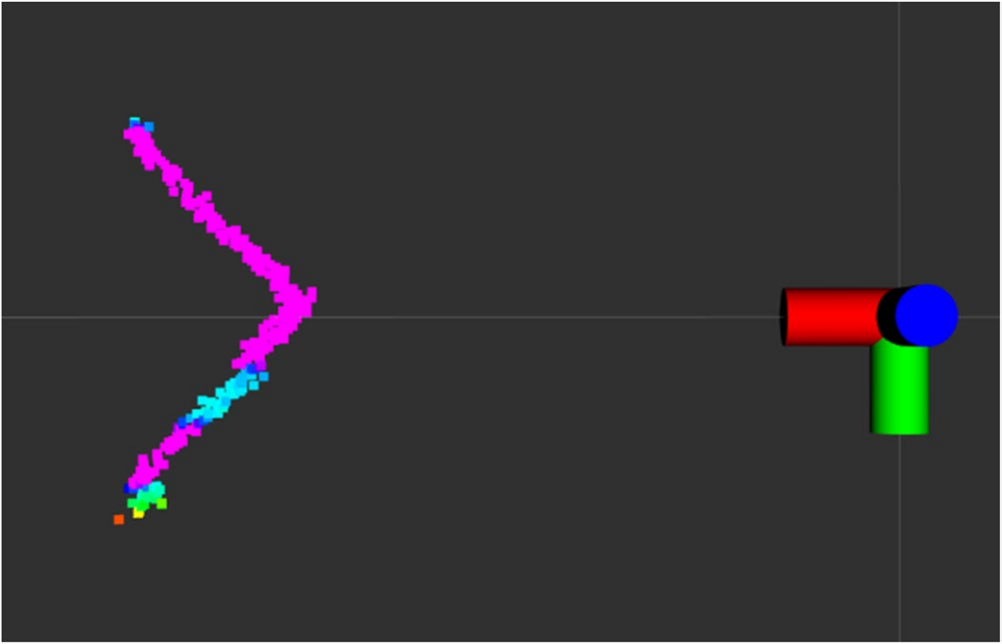
The 2DM-RC laser scan that was obtained by the industrial 2D LiDAR Sick microscan3.

Figure 8 shows how the 2D LiDAR Sick microscan3 sees the test 2DM-RC that is perceived by its laser beams. The pinkish colour is the more reflective surface, and the blueish one is less reflective.

Listing 1 presents the example ROS message that was printed out after the left AS leg (2DM-RC with 101 binary = ID 5) was detected and identified. The message contains the information about the position and orientation of the AS leg (of ROS geometry_msgs/Pose type).

**Listing 1:**
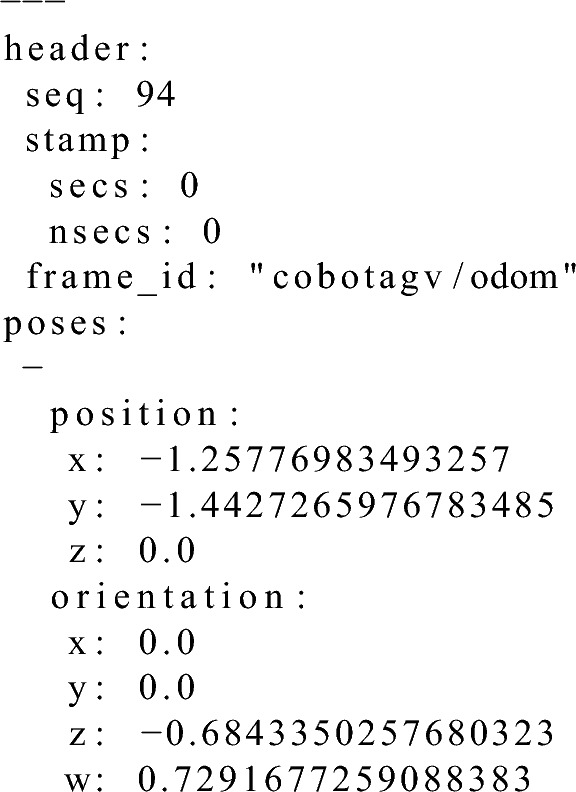
Example message about the detected AS leg (position and orientation) on the /assembly_station_legs ROS topic.

### The method of calculating the docking point pose

**Figure 9:**
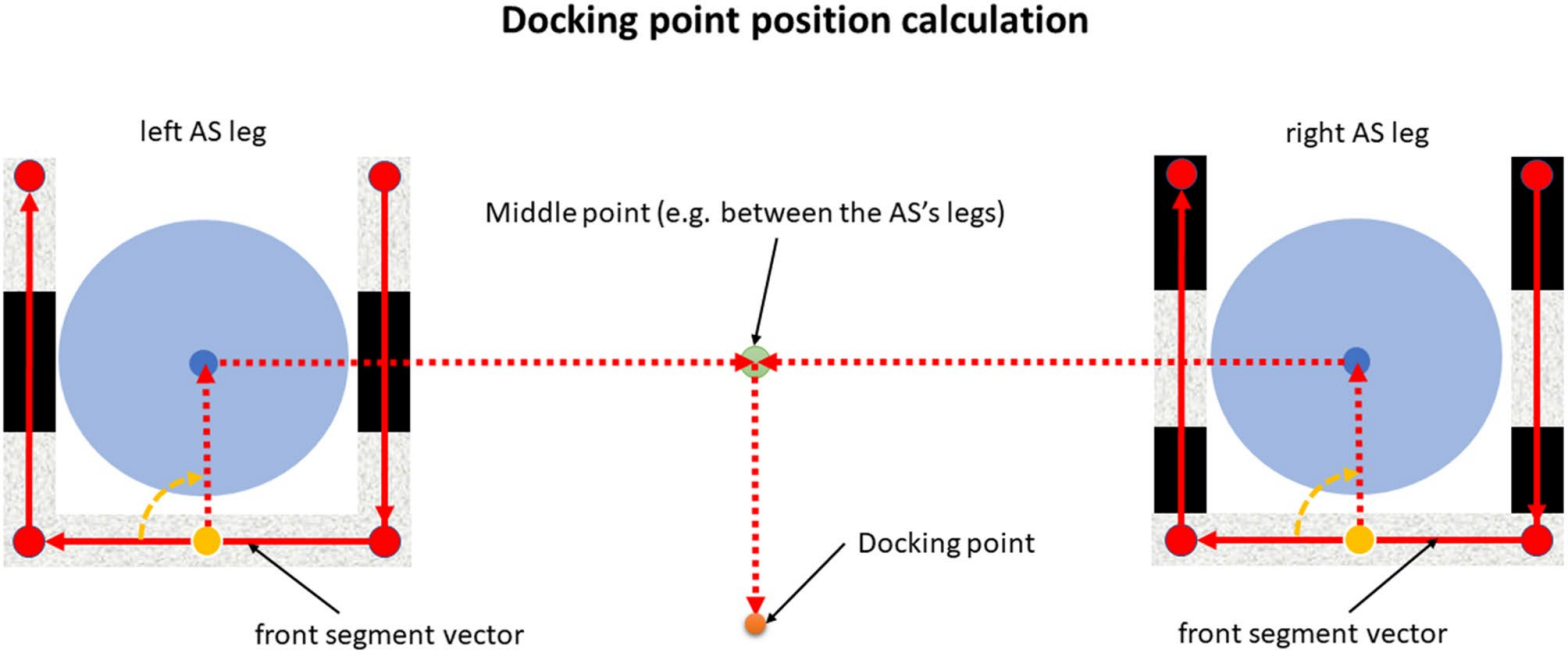
Visualisation of the docking point position calculation method for an AS equipped with the 2DM-RC mounted on the legs.

The docking point is calculated using the information about the position of the AS legs and the 2DM-RCs segments that were denoted as the front side (ID=7) (Fig. 9).

Firstly, the vectors are calculated knowing the start and end points of the front segments. Then, the vector of the left leg of the AS was rotated 180 degrees. Both vectors were normalised and multiplied by the distance from each of the legs to the point corresponding to the docking point. These vectors were added to the positions of the legs of the AS, which was calculated as the middle point between the AS legs. Then, the vectors were normalised and rotated once again by 90 degrees, the vector from the segment of the left leg clockwise and the vector from the segment of the right leg counterclockwise. These rotated vectors were multiplied by the desired distance from the AS legs to the docking point and added to the previously calculated points between the AS legs. The result of these operations were two positions whose mean gave the docking point position. The orientation of the docking point is the mean of the front segments of the AS legs rotated 180 degrees. The docking point position and orientation is calculated every time an AS leg is detected but the docking point that is used in the docking and verification phases is calculated as the moving average of the last five detections.

This calculated docking point pose is published to the ROS environment and is received by the action client who sends the goal position and orientation to the ROS Navigation Stack, which takes control of the CoBotAGV and drives it to the calculated docking point position.

### Development of verification method for estimating the AGV to AS relative pose

Based on the research for 2D reflection-based marker detection and identification method, docking algorithm and ROS platform functionalities, there is a way to perform the verification phase in order to calculate the correction value after the CoBotAGV has docked in the desired docking point. The verification phase enables the CoBotAGV position and orientation errors after the docking phase to be verified. Accurately measuring the errors and knowing their values enables actions to be taken in order to eliminate them (especially if the docking accuracy error is close to the assumed maximum of 2 cm), e.g., by other subsystems that can counteract the errors such as the robot or collaborative robot arm.

The AGV platforms have a specific description in the ROS environment that is called a Unified Robot Description Format (URDF). The defined links on an AGV are a set of XML entries that describe the dimensions of the robot and the position (X,Y,Z) and orientation of its wheels, LiDAR etc. in relation to the middle point of the robot platform, which is called the “base_link” frame. There can be multiple frames located on the robot, e.g., the “base_footprint” a frame that is identical to the “base_link” frame except that it does not provide any information in the Z axis and can be seen as a projection of the “base_link” on the floor.

Such an approach enables to calculate the relative distance and orientation of specific point in the AGV environment to the one of the elements of the AGV platform. In order to calculate the translations between two points in the AGV’s environment from the ROS platform, there is the tf2 package^46^, which stands for the transformation. The functions that this package contains take as an input the source and the target frames in order to calculate a translation between these two coordinate systems. Then, the calculated translation can be used to calculate where any point from the source coordinate system (source frame) is located in the target coordinate system (target frame). Precise information about the transformation (position and orientation) between two frames can be calculated using the tf2_ros::Buffer::lookupTransform() function in the C++ code.

The desired position and orientation of the docking point in an AGV’s environment coordinate system is known as well as the position and orientation of the AGV itself and the position and orientation of the AS. With that information, it is possible to calculate the correction value (error of docking) in the verification phase (Fig. 6) using the methods provided in the tf2 ROS package (Algorithm 1).

Listing 2 presents the calculated docking point pose, Listing 3, the position that was reached by the AGV and Listing 4, results of the calculations (using Algorithm 1) between these two the correction in X, Y-axis and the shortest yaw angle correction that would have to be made in order to be in the exact desired docking point position and orientation.

**Listing 2:**
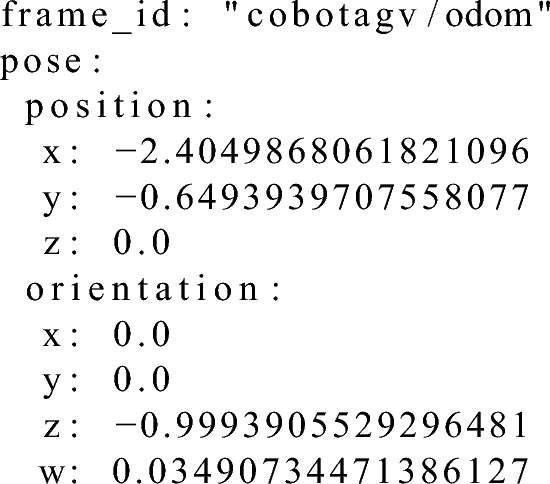
Example input data of the desired docking point pose for the correction calculation algorithm.

**Listing 3:**
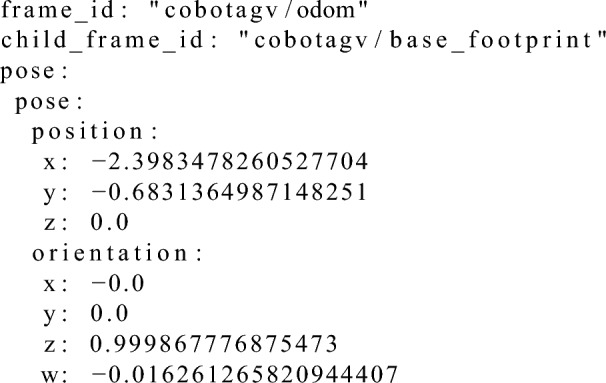
Example input data of the Formica 3 CoBotAGV pose for the correction calculation algorithm.

**Listing 4:**
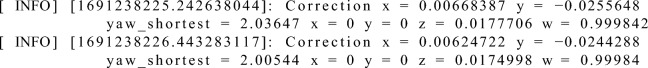
Example output from the algorithm to calculate the correction values during verification phase. (Correction forrespectively X and Y axes positions shortest yaw orientation angle and orientation in quaternion).

The verification phase, which is responsible for calculating the correction value after the AGV has docked, can be summarised as a loop with the following steps:

1. Locate the AS elements using the developed 2DM-RC detection and identification method,
2. Calculate the docking point knowing the position and orientation of the AS’s elements makes it possible to calculate the docking point position and orientation after having specified the desired distances of the docking point from the AS’s elements Fig. 6,
3. Calculate the difference between the AGV’s position and the calculated position of the docking point in the $2^{\text {nd}}$ step,
4. Transform (rotate) the calculated difference in $4^{\text {th}}$ point by the inverse of the desired docking point orientation from $2^{\text {nd}}$ step,
5. Calculate the shortest difference between the desired docking point orientation and the AGV’s orientation.

The final correction value consists of results from the $4^{\text {th}}$ and $5^{\text {th}}$ points, which are the linear (X,Y) and angular (YAW) corrections, respectively. The X-axis is parallel to the line between the left and right legs of the AS and the axis direction goes from the left to right leg. The Y-axis is perpendicular to the line between the left and right legs of the AS and the axis direction goes up towards the AS.

**Algorithm 1:**
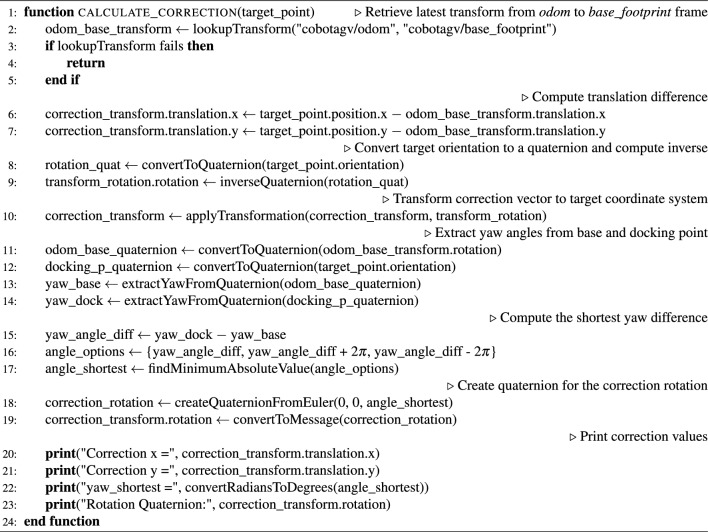
Docking point correction calculation during the verification phase

## Experiments to investigate the quality of the AGV docking

The docking and verification experiments were conducted as described in previous section using the Formica 3 CoBotAGV. Both phases were based on the developed detection and identification method of 2D reflection-based markers. The Formica 3 is a heavy industrial AGV platform (0.85x0.58m) that is designed to lift and transport goods in a warehouse. The Formica3 CoBotAGV has two motors, one on each of its sides and uses the differential control model.

**Figure 10:**
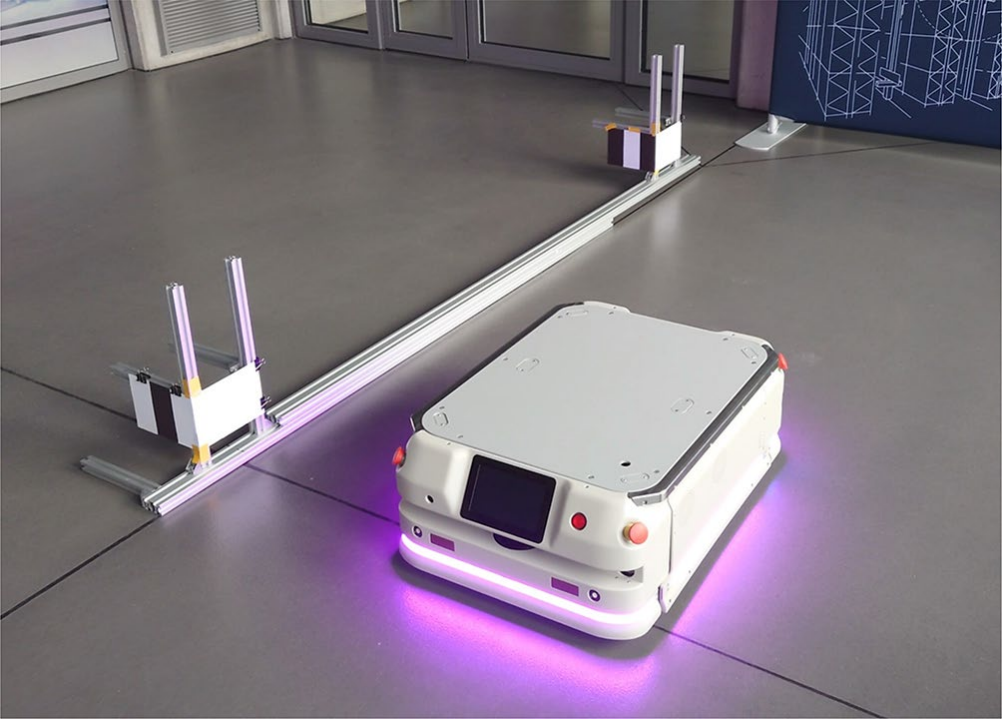
Testbed for Formica 3 CoBotAGV docking experiments to 2DM-RCs mounted on the left and right legs of the AS.

In the experimental description, the term “docking/verification to the left/right leg” denotes that the docking point position and orientation calculation was conducted using the 2DM-RC that was mounted on the left or right leg of the AS. The AS docking research station (Fig. 10) consisted of aluminium rails on which the two 2DM-RCs were mounted (for the left and right legs). The AGV began its docking phase from the left side of the AS research stand and about 2m away from it. After the identification of the left 2DM-RC and calculation of the docking point position and orientation, the AGV proceeded along its route. Then, the AGV docked to the left leg of the AS and verified its position in relation to the legs. The verification process enables the final docking position of the AGV to be assessed.

The experiment was conducted using Sick microscan3 2D LiDAR scanners, which were mounted in the rear and front of the AGV. The adaptive calibration method^47^ was used to calibrate the distance measurements from the 2D LiDAR, which ensured accurate measurements.

The docking experiments, which were followed by the verification of the docking quality, were performed in three variants. Each docking and verification experiment was conducted using calibrated 2D LiDARs. Additionally, each variant had its own conditions as described below:

1. Docking into the left leg of the AS and verification to the right leg
  - lack of an odometry error correction
2. Docking into left leg of the AS and verification to both legs
  - with an attempt to correct the odometry error (angular error of -0.054 rad/m)
3. Docking into left leg of the AS and verification to both legs
  - with an attempt to correct the odometry error (angular error of -0.054 rad/m)
  - correction of the docking error on the Y-axis (based on the mean error from the $2^{\text {nd}}$ variant)

### A summary of the quality of the AGV docking

Table 1 presents the docking variants and their conditions. In each of the variants presented in Tables 1 and 2, the docking results were reproducible, and the results of the verification process were comparable. Table 2 presents the mean errors (ME)s, Euclidean MEs and standard deviations (STDEV)s of the AGV docking attempts, where the errors represent differences from the measured and calculated target docking point. These errors can be categorized into three major parts: the errors of docking that were measured using a measuring tape, the errors of docking that were measured during the verification process and the difference between these two. The results in Table 2 allow an assessment of the accuracy and precision of the docking to the measured and calculated docking point for 2DM-RC mounted on the left and right AS legs.

**Table 1:** Variants of docking an AGV to an AS.

No.	Docking to ... leg	Verification to ... leg	2D LiDAR calibration	Odometry correction	ME correction for Y-axis
1.	Left	Right	+	−	−
2.	Left	Both	+	+	−
3.	Left	Both	+	+	+

As the results for the $1^{\text {st}}$ docking variant show, the AGV was docking far too close to the AS and its relative orientation was turned to the left. The Euclidean mean distance error was 0.1794 m (0.0015 m on the X-axis, − 0.1794 m on Y-axis) and the mean YAW orientation was − 7.32 degrees. The correction values / errors that were calculated during the verification process differed from the ones that were measured manually by 0.0264 m for the Euclidean ME (− 0.0156 m on the X-axis and 0.0213 m on the Y-axis) and by a mean of 1.29 degrees in the YAW orientation.

The results for the $2^{\text {nd}}$ variant present the AGV docking assessment that introduced an attempt to correct the odometry error (mean error of − 0.054 rad/m). As the results show, after the odometry correction, the Euclidean mean distance error decreased to 0.0449 m (0.0053 m on the X-axis and − 0.0446 m on the Y-axis) and the mean YAW orientation error decreased to 0.076 degrees. During the $2^{\text {nd}}$ variant of docking, two 2DM-RCs were used for the verification process. The correction values / errors calculated during the verification process differed from the manually measured ones by 0.0049 m for Euclidean ME (− 0.001 m on the X-axis and − 0.0048 m on the Y-axis) and by 0.58 degrees in the YAW orientation.

The results for the $3^{\text {rd}}$ variant present the AGV docking assessment with the correction (4.46 cm) on the Y-axis. The results shows that the Euclidean mean distance error decreased to 0.0097 m - on the Y-axis it decreased to -0.0055 m while on the X-axis, it increased to 0.008 m. The verification process also accurately calculated the error of the AGV position and orientation at the final docking point. The mean differences between the errors that were measured manually and the ones that were calculated during the verification process were 0.0058 m for Euclidean ME (0.0058 m on the X-axis, − 0.0007 m on the Y-axis) and 0.21 degrees for the YAW orientation. These results prove that the accuracy error correction had an influence on the final docking accuracy.

The $2^{\text {nd}}$ variant (where the odometry correction was performed) when compared with the $1^{\text {st}}$ variant decreased the accuracy by 3.8 mm from the 0.0015 m to the 0.0053 m for the X-axis but increased the accuracy from the − 0.1794 m to the − 0.0446 m for the Y-axis and from − 7.3203 degrees to the -0.0759 degrees for the YAW orientation. The final experiment was conducted with the correction on the Y-axis and then compared to the $2^{\text {nd}}$ experiment; the correction enabled the accuracy to be increased from − 0.0446 m to − 0.0055 m for the Y-axis and from − 0.0759 to − 0.0473 degrees for the YAW orientation.

What is also important and worth mentioning is that the verification process was accurate and enabled the errors of the docking process to be detected and calculated, e.g. during the $2^{\text {nd}}$ experiment, the measured errors for the X, Y and YAW orientation were 0.0053 m, − 0.0446 m and − 0.0759 degrees, respectively and the errors for the X, Y and YAW orientation that were calculated during the verification process were 0.0063 m, − 0.0398 m and 0.5099 degrees, respectively. With the information about the docking errors from the verification process, it is possible to minimise the final docking error to the level of the difference between the manually measured errors and the ones that were calculated during the verification process, which averaged 1 mm on the X-axis, 4.8 mm on the Y-axis and 0.585 degrees in the YAW orientation.

**Table 2:** Result of CoBotAGV docking accuracy and precision to the measured and calculated docking point for 2DM-RC mounted on the right and left AS legs.

Var. no.	Metrics	The manually measured distances with a measuring tape			The automatically measured distances with the 2D LiDAR during the verification process			Differences between the manually and automatically measured distances		
		X [m]	Y [m]	YAW [deg.]	X [m]	Y [m]	YAW [deg.]	X [m]	Y [m]	YAW [deg.]
1.	ME	0.0015	− 0.1794	− 7.3203	0.0171	− 0.2007	− 6.0278	− 0.0156	0.0213	− 1.2926
	Euclidean ME	0.1794		–	0.2014		–	0.0264		–
	STDEV	0.0087	0.0194	2.0770	0.0058	0.0289	1.8347	0.0029	− 0.0095	0.2423
2.	ME	0.0053	− 0.0446	− 0.0759	0.0063	− 0.0398	0.5099	− 0.0010	− 0.0048	− 0.5858
	Euclidean ME	0.0449		–	0.0403		–	0.0049		–
	STDEV	0.0058	0.0117	1.7348	0.0068	0.0105	1.8563	− 0.0011	0.0012	− 0.1215
3.	ME	0.0080	− 0.0055	− 0.0473	0.0022	− 0.0048	0.1699	0.0058	− 0.0007	− 0.2172
	Euclidean ME	0.0097		–	0.0053		–	0.0058		–
	STDEV	0.0120	0.0191	1.6740	0.0102	0.0153	1.6192	0.0018	0.0038	0.0548

The Table 3 presents the quality assessment of docking for all of the tested variants, but the errors for the X and Y axes are the sums of the ME for a given axis and its STDEV. Thus Table 3 presents the maximum errors for both axes and orientation as shifts from desired docking point positions. For each variant and its errors, the maximum Euclidean error was calculated. Based on Table 3, the most accurate and precise variant of the docking (without the error correction on the Y-axis) was the $2^{\text {nd}}$ variant, which had the smallest maximum Euclidean distance and the smallest error during the verification process. In this case, the smallest differences were obtained between the distance errors that were measured with a measuring tape and with the 2D LiDAR during verification process, 0.001 m for the X-axis, 0.0048 m for the Y-axis and 0.5858 degrees for the YAW. However, the $3^{\text {rd}}$ variant achieved the smallest Euclidean error for both the manually and automatically measured errors (during the verification phase) due to the correction on the Y-axis.

As a result, 2D LiDAR can be used to verify the position of an AGV after docking to the 2DM-RCs that are mounted on an AS with a maximum measurement error of 0.0021 m for the X-axis, 0.0060 m for the Y-axis and 0.7073 degrees for the YAW and a maximum Euclidean distance of 0.0064 m. Similar results were confirmed in the $3^{\text {rd}}$ variant where in this case, the difference between the manually measured error and the one that was measured during verification was 0.0076m for the X-axis, 0.0045m for the Y-axis and 0.272 degrees for the YAW orientation. In effect, the developed methods of docking using the developed markers enabled the Formica3 AGV for a docking accuracy of below the 2 cm that were assumed in the project CoBotAGV to be obtained.

**Table 3:** Maximum shifts from the desired docking points positions after docking attempts.

Var. no.	Max. shift	The manually measured distances with a measuring tape			The automatically measured distances with the 2D LiDAR during the verification process			Differences between the manually and automatically measured distances		
		X [m]	Y [m]	YAW [deg.]	X [m]	X [m]	YAW [deg.]	X [m]	Y [m]	YAW [deg.]
1.	Avg. + STDEV	0.0102	0.1988	9.3973	0.0229	0.2295	7.8625	0.0185	0.0307	1.5349
	Max. Euclidean distance	0.1991		–	0.2307		–	0.0359		–
2.	Avg. + STDEV	0.0111	0.0563	1.8107	0.0132	0.0503	2.3662	0.0021	0.0060	0.7073
	Max. Euclidean distance	0.0573		–	0.0520		–	0.0064		–
3.	Avg. + STDEV	0.0200	0.0246	1.7213	0.0124	0.0201	1.7891	0.0076	0.0045	0.2720
	Max. Euclidean distance	0.0317		–	0.0236		–	0.0088		–

### Experimental verification of the quality of the detection of the reflection-based 2D markers

**Figure 11:**
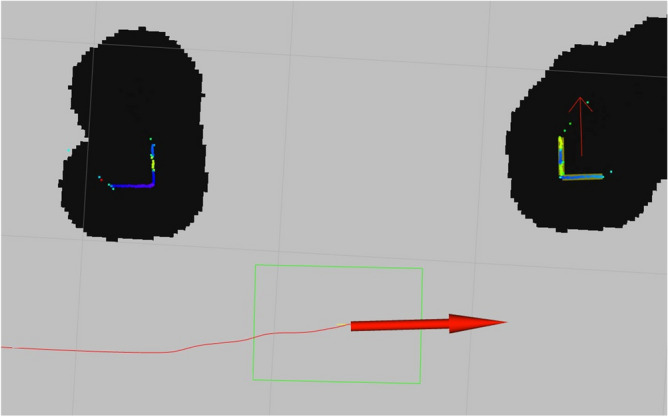
The view of the two 2DM-RCs mounted on the AS legs and the calculated docking point, from the front and rear 2D LiDAR mounted on the AGV.

The objective of this study was to provide an experimental verification of the quality of the detection of reflection-based markers. Before the docking process begins and during the verification process, an AGV looks for the 2DM-RCs in the LiDAR scans. During the docking and verification experiments, the data of 2DM-RCs’ (mounted on the AS legs) position and orientation were gathered and processed. Figure 11 shows the AGV (green rectangle) near the docking point (horizontal red arrow) between the two AS legs, which have the 2DM-RCs. The red line shows the planned route to the docking point, which is calculated by the ROS Navigation Stack after the detection and identification of the 2DM-RCs.

The vertical segments of the 2DM-RCs identify the leg (left or right) and the horizontal part has a common segment identifier for both 2DM-RCs and helps in calculating the orientation of the legs. The vertical part consists of the subelements (the more and less reflective parts), which create the 3-bit structure that enable the legs to be identified. The red arrow pointing up (in the middle of the right 2DM-RC) is the middle position of the right leg and its orientation corresponds to the Y-axis of the AS coordinate system, and it is perpendicular to the front part (horizontal segment) of the leg and to the desired docking point orientation.

Table 4 contains the analysis of the two (left and right) legs and shows the analysis of the positioning of the AS legs in the X,Y axes and its orientation. During the verification process, both legs were seen by the AGV, which had two LiDARs (rear and front). The mean STDEV for the two 2DM-RCs (left and right) differed by 0.8 mm for the Euclidean distance the front 2D LiDAR had higher STDEV of 0.0034 m of the right leg position. The difference was equal to the STDEV of the AS leg position STDEV values. On the other hand, the front 2D LiDAR had a smaller STDEV of the right AS leg YAW orientation by 0.0357 degree than the rear 2D LiDAR, but the difference was smaller than the STDEV of the orientation STDEV values.

The differences in STDEVs of the 2D LiDARs might have been caused by their own measurement characteristics. The STDEV of both 2D LiDARs corresponded with the measurement characteristics of the Sick microscan3 2D LiDAR scanner^48^ its mean STDEV was 2.5 mm. Thereby it is worth mentioning that the precision of the positioning equalled the precision of the 2D LiDAR itself.

**Table 4:** The verification of the positioning precision of AS legs with the mounted 2DM-RCs.

No.	Assembly station leg pose STDEV					
	LEFT LEG rear 2D LiDAR			RIGHT LEG front 2D LiDAR		
	X [m]	Y [m]	YAW [deg]	X [m]	Y [m]	YAW [deg]
1.	0.0011	0.0026	0.1844	0.0014	0.0026	0.2484
2.	0.0011	0.0013	0.3098	0.0012	0.0017	0.2365
3.	0.0025	0.0023	0.3651	0.0035	0.0037	0.1237
4.	0.0010	0.0017	0.2128	0.0016	0.0030	0.2941
5.	0.0009	0.0017	0.1348	0.0016	0.0039	0.3032
6.	0.0020	0.0014	0.8248	0.0012	0.0021	0.3499
7.	0.0015	0.0023	0.2590	0.0016	0.0029	0.2178
8.	0.0008	0.0028	0.2026	0.0024	0.0033	0.2395
9.	0.0029	0.0025	0.2824	0.0013	0.0022	0.2518
10.	0.0013	0.0026	0.5100	0.0019	0.0027	0.3517
11.	0.0012	0.0020	0.1962	0.0011	0.0043	0.3617
12.	0.0014	0.0022	0.2402	0.0011	0.0034	0.3156
Mean	0.0015	0.0021	0.3102	0.0017	0.0030	0.2745
STDEV	0.0007	0.0005	0.1899	0.0007	0.0008	0.0687
Mean Euclidean distance	0.0026		–	0.0034		–

## Conclusions

Some 2D LiDARs are capable of measuring varying levels of reflected light strength, which makes it possible to design markers that encode binary values by altering surface reflectivity. Experimental tests demonstrated that these properties can be effectively exploited to detect and identify reflection-based markers using a 2D LiDAR capable of measuring the strength of the reflected laser beam. Marker identification is performed by first recognizing its geometric shape, after which its pose (position and orientation) can be determined. Using the developed 3-bit marker structure, objects were successfully identified by decoding the embedded binary values. Furthermore, the method can be extended to multi-bit 2DM-RC tags with different number of bits. Since the approach relies on reflectivity measurements, its performance is inherently sensitive to surface conditions. The impact of surface conditions variability on robustness was not examined in this study but will be addressed in future research.

The developed 2DM-RC and its detection and identification method were successfully applied in the AGV docking scenario and subsequently used in the verification phase, which was essential for assessing docking quality. The verification process enabled the measurement of docking errors, and the estimated values closely matched those obtained through manual measurements. Moreover, the proposed 2DM-RC and detection method can be integrated into any autonomous platform equipped with 2D LiDAR technology.

Due to the developed docking methods, which combine the 2DM-RC with precise distance measurements from calibrated 2D LiDARs, it was possible to achieve accuracy up to 1 cm and less than 0.05 degree for the YAW orientation, as confirmed by manual measurements. It is also worth mentioning that the STDEV in most cases was higher than the mean accuracy error. However, the mean of accuracies of the docking or verification processes were high considering that the platform that was used for the experiments was a prototype of the heavy industrial CoBotAGV whose size many times exceeds the value of the accuracy error.

What is even more important than accurate docking is the ability to determine errors accurately and precisely due to the verification process, which is based on 2DM-RCs. Knowing the accuracy error gives a user the possibility to apply a correction, otherwise (without the verification process) it would not be possible to correct it. It was demonstrated that, after calibration, Sick microscan3 2D LiDAR verified the position with the smallest Euclidean ME of 0.0049 m (-0.001 m for the X-axis and -0.0048 m for the Y-axis) and 0.5858 degrees for the YAW orientation, which was calculated based on the differences between the distance errors measured with a measuring tape and those obtained from the 2D LiDAR during the verification process.

It should be mentioned that the precision and accuracy of the positioning depended on the precision and accuracy of the 2D LiDAR itself and the quality of its mounting on the AGV. Consequently, using two or more 2D LiDARs would require further development of methodologies for the calibration of the position and orientation within the context of the AGV system.

## Data Availability

The datasets generated and analysed during this study are available from the corresponding author upon reasonable request.
